# Transforming Growth Factor Beta Signaling in Dendritic Cells Is Required for Immunotolerance to Sperm in the Epididymis

**DOI:** 10.3389/fimmu.2018.01882

**Published:** 2018-08-16

**Authors:** Fernando Pierucci-Alves, Monica T. Midura-Kiela, Sherry D. Fleming, Bruce D. Schultz, Pawel R. Kiela

**Affiliations:** ^1^Department of Anatomy and Physiology, Kansas State University, Manhattan, KS, United States; ^2^Department of Pediatrics, University of Arizona, Tucson, AZ, United States; ^3^Division of Biology, Kansas State University, Manhattan, KS, United States; ^4^Department of Immunobiology, University of Arizona, Tucson, AZ, United States

**Keywords:** sperm tolerance, infertility, autoimmunity, transforming growth factor, dendritic cells

## Abstract

The epididymis exhibits a less restrictive physical blood–tissue barrier than the testis and, while numerous immunosuppressive factors have been identified in the latter, no mechanisms for epididymal immunotolerance have been identified to date. Therefore, data are currently insufficient to explain how the immune system tolerates the extremely large load of novel antigens expressed on sperm, which become present in the male body after puberty, i.e., long after central tolerance was established. This study tested the hypothesis that transforming growth factor beta (TGFβ) signaling in dendritic cells (DCs) is required for immunotolerance to sperm located in the epididymis, and that male mice lacking TGFβ signaling in DCs would develop severe epididymal inflammation. To test this, we employed adult *Tgfbr2*^ΔDC^ males, which exhibit a significant reduction of *Tgfbr*2 expression and TGFβ signaling in DCs, as reported previously. Results show that *Tgfbr2*^ΔDC^ males exhibit sperm-specific immune response and severe epididymal leukocytosis. This phenotype is consistent with epididymal loss of immunotolerance to sperm and suggests that TGFβ signaling in DCs is a factor required for a non-inflammatory steady state in the epididymis, and therefore for male tract homeostasis and function.

## Introduction

The epididymis is already known for contributing directly to male reproductive function by promoting sperm maturation and serving as storage site for sperm (1). The epididymal duct is lined by pseudo-stratified epithelia that form tight junctions and implement, among other important factors, a physical barrier. Importantly, sperm are immunogenic (2, 3) and become present in the body long after central (thymic) immunotolerance is established. The physical testicular barrier and testicular immunosuppressive factors (4, 5) are thought to maintain a non-inflammatory local steady state that is conducive to reproductive function. However, the epididymis exhibits a less restrictive physical barrier (6) and no active immunosuppressive mechanisms to prevent antisperm immunity have been identified in this organ (7). Abundant mononuclear phagocytes (MPs) populate the epididymis and dendritic cells (DCs)—characterized by CD11c expression, typical DC morphology, and antigen presentation activity *in vitro*—constitute a substantial MP population present in the epididymis at steady state (8). DCs link innate and adaptive components of the immune system, by either initiating immune responses or inducing immunotolerance (9). Physiological roles for epididymal DCs have not yet been identified.

Transforming growth factor beta (TGFβ) isoforms 1, 2, and 3 are ligands that activate the same receptor complex (TGFβ receptors I and II) to induce signal transduction through canonical and non-canonical mediators (10–14). Although TGFβ isoforms are known to exert pleiotropic effects (15, 16), their immunological regulatory function is well established. TGFβ1 is an immunosuppressive cytokine as demonstrated *in vivo* with the global *Tgfb1*-null mouse (17, 18), and subsequently with a mouse carrying T cell-specific suppression of TGFβ signaling (19). Importantly, the male reproductive tract synthesizes and secretes large amounts of TGFβ1: median concentrations of total and active TGFβ1 in seminal plasma are 85 and 1 ng/ml, respectively (20). Although seminal plasma is largely a product of seminal vesicle secretion, gene and protein expression data suggest that the epididymis and vas deferens also express TGFβ isoforms and receptors (21–24).

This study tested the hypothesis that TGFβ signaling in DCs is required for immunotolerance to sperm, and that male mice lacking TGFβ signaling in DCs would develop severe epididymal inflammation. To test this, we employed adult *Tgfbr2*^ΔDC^ males, which exhibit a significant reduction of *Tgfbr*2 expression and TGFβ signaling in DCs (25). Our results show that *Tgfbr2*^ΔDC^ males exhibit sperm-specific immune response and epididymal leukocytosis that can reach extremely severe stages. This phenotype is consistent with loss of immunotolerance to sperm and suggests that TGFβ signaling in DCs is a factor required for a non-inflammatory steady state in the epididymis, and therefore for male tract homeostasis and function.

## Materials and Methods

### Mice

*Tgfbr2*^ΔDC^ mice (25) carrying a FoxP3-GFP knock-in allele (26) were generated and utilized under the guidelines of an animal protocol approved by the Kansas State University Institutional Animal Care and Use Committee (IACUC). *Tgfbr2*^ΔDC^*Rag2^−/−^* mice were generated and utilized as approved in an University of Arizona IACUC approved animal protocol.

### Histology

Male excurrent system and testicular tissues from *Tgfbr2*^ΔDC^, *Tgfbr2*^ΔDC^*Rag2^−/−^*, and littermate control (*Cre*^−^) males aged 8–10 weeks were fixed in block by immersion in Bouin’s solution. Dissected epididymis and testis were blocked in paraffin and sectioned at 6 µm. Epididymal tissue sections were made parallel to the long axis of the organ to include both the proximal and distal epididymal segments. Testicular sections were made across the long axis. Sections were stained with hematoxylin and eosin and slides (one per organ) were analyzed and photographed with an Eclipse E600 microscope and DS-Vi1 camera head (Nikon).

### Flow Cytometry

Epididymal, testicular, and renal tissues were processed for flow cytometry as recently reported in a peer-reviewed protocol optimized for non-lymphoid murine tissues (27). Testicular tissue was completely dissociated after 45–60 min of incubation in the enzymatic step of this protocol, while epididymal tissue required 60–75 min. Splenocyte suspensions were obtained by mechanical dissociation of scissors minced spleen in Hank’s balanced salt solution containing HEPES (10 mM) and fetal bovine serum (5%). All cell suspensions were strained (70 µm), washed, and counted by the standard hemocytometer method prior to flow cytometry staining, which was conducted as reported previously (27). The fixable cell viability dye eFluor 506 (eBiosciences) was employed as per the manufacturer’s recommendations. The flow cytometry antibodies employed were PE-Cy5 anti-mouse CD45 (clone 30-F11, eBiosciences), PE-Cy5 rat IgG2b, κ isotype antibody control (clone eB149/10H5, eBiosciences), APC anti-mouse CD25 (clone PC61, BioLegend), and APC rat IgG1, λ isotype antibody control (clone G0114F7, BioLegend). Following staining, cells were washed, fixed in 0.4% paraformaldehyde in PBS, and stored at 4°C protected from light until data acquisition (24–48 h), as reported previously (28). Compensation samples were UltraComp compensation beads (eBiosciences) stained with antibodies or splenocytes stained with the viability dye as per the manufacturer’s recommendations or unstained splenocytes, which contained the green fluorescent protein (GFP) signal in regulatory T cells (T_regs_). Data were acquired in a LSR Fortessa X20 (BD Biosciences) and were compensated and analyzed in FCS Express (*De Novo* Software, v6.05). In data analyses, CD45^+^ gates were placed individually for each tissue type and experimental group so that 0.3% of the isotype control stained cells were allowed in the gate. Gate placement was constant across the different tissues measured. Isotype control values were subtracted from CD45 antibody-specific stained values on a % basis. FCS Express built-in statistical tokens were employed in a built-in spreadsheet to tabulate outcomes for each tissue and experimental group and in each experiment. Data in built-in spreadsheets were then exported, compiled, and analyzed statistically.

### Magnetic Bead Cell Sorting

Cell suspensions were generated identically to that done for flow cytometry and then subjected to T cell (CD4^+^) magnetic bead sorting as per the manufacturer’s recommendations (Stemcell). Sorted isolates were subjected to flow cytometry as described above.

### Antisperm Antibody (ASA) Detections

To detect ASAs bound to sperm, flow cytometry was employed. Immediately after the enzymatic step and cell suspension straining in the flow cytometry protocol, samples were centrifuged at 300 *g* for 5 min. Epididymal samples consistently produced a cloudy supernatant that was rich in sperm, as visualized in a hemocytometer. Sperm in this supernatant were recovered by additional centrifugation at 600 *g*, and subjected to the flow cytometry staining procedure described above. Cell viability dye was not employed. For any given sperm sample, cells were either not incubated with staining antibody or incubated with Alexa Fluor 488 anti-mouse IgG or Alexa Fluor 594 anti-mouse IgG (Invitrogen). To detect ASAs in sera, an immunoblot-based assay was conducted. Sperm were isolated from cauda epididymis of non-transgenic C57B6 males by the swim out method in Whittens-HEPES medium and were solubilized in Celis buffer as described previously (29). Approximately 200 µg of total sperm protein was resolved on single-lane IPG gels (SDS-PAGE, 10%), which were blotted onto nitrocellulose membranes. Membranes were washed and blocked with 5% non-fat dry milk powder in TBS containing 0.05% Tween-20 (TBST) for 1 h, and then mounted on a Mini-PROTEAN II Multiscreen Apparatus (Bio-Rad). Each serum sample tested was diluted at 1:100 in TBST, and this preparation was applied to a dedicated well in the apparatus for incubation at 4°C overnight. Serum preparations were removed from each well, which were washed with TBST and then replenished with HRP-conjugated anti-mouse IgG at 1:5,000 in TBST. Following incubation for 1 h at room temperature, membranes were unmounted, washed, incubated with Supersignal West Pico HRP substrate (Pierce, IL, USA), and imaged.

### Gene Microarray Analysis

RNA was isolated using TRIZOL (Thermo Fisher Scientific) and further purified with the RNeasy PowerClean Pro CleanUp kit (Qiagen). RNA integrity analysis (Bioanalyzer 2100, Agilent) resulted in RIN ≥ 9.0, and RNA concentrations were determined with Nanodrop (Thermo Fisher Scientific). Amplified and biotinylated sense-stranded DNA targets were generated from each of 12 unique RNA samples derived from *Cre*^−^ and *Tgfbr2*^ΔDC^ epididymides and testes (*n* = 3 in each experimental group) using the GeneChip WT PLUS Reagent kit (Affymetrix), and subsequently hybridized to GeneChip Mouse Gene 2.0 ST arrays (Affymetrix). Microarray data analyses were performed using GeneSpring (Agilent, v. 14.9). Data were processed using the RMA16 summarization algorithm and normalized against the mean of control (*Cre*^−^) samples for visualization. Statistical analysis was performed using built-in tools, including moderated *t*-test (*p* ≤ 0.05) with Westfall–Young multiple testing correction. Gene ontology functional annotation analyses were performed with either GeneSpring or the online tool made available by Database for Annotation, Visualization, and Integrated Discovery (v6.8, National Cancer Institute at Frederick) (30, 31). Ingenuity Pathway Analysis (IPA) (32) (Qiagen) was conducted to identify and compare overrepresented pathways affected in *Tgfbr2*^ΔDC^ epididymides and testes. Additional analyses outcomes, including raw and normalized expression values, can be viewed at the National Center for Biotechnology Information Gene Expression Omnibus microarray depository website (GEO accession no. GSE118262).

### Statistical Analyses, Figure Preparation, and Data Availability Statement

Flow cytometry plots were made with FCS Express, while numerical data were compiled in Excel (Microsoft), which was used to calculate means, SEM, and paired *t*-tests. Bar graphs with SEM were generated in SigmaPlot (Systat Software, v. 6.0) and figures were made in CorelDRAW 2017 (Corel Corporation, v19.1). The raw data supporting the conclusions of this manuscript will be made available by the authors, without undue reservation, to any qualified researcher.

## Results

### TGFβ-Signaling Loss in DCs Leads to Severe Epididymal Leukocytosis

**Figure 1:**
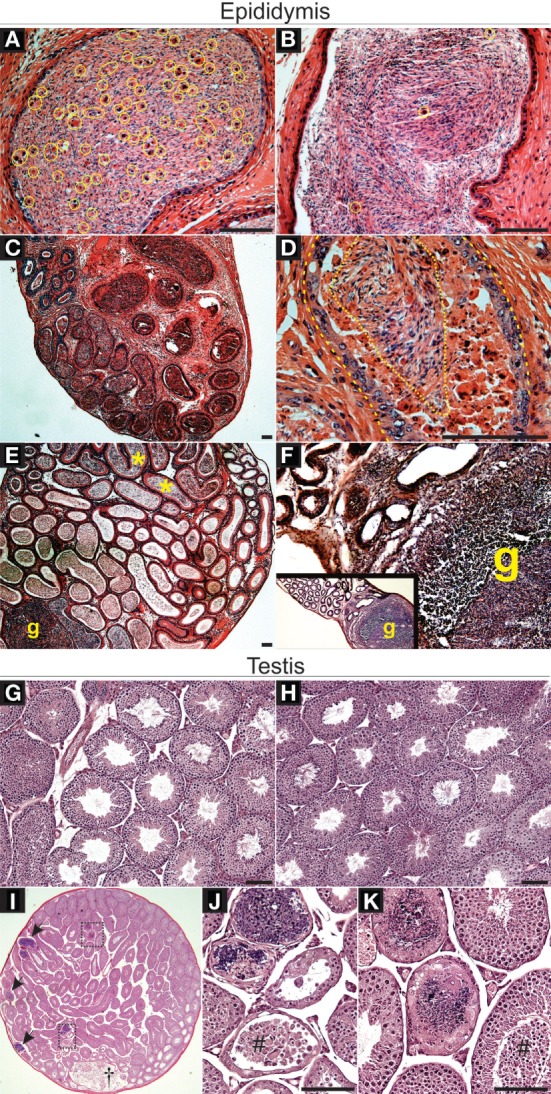
Loss of transforming growth factor beta (TGFβ)-signaling in dendritic cells (DCs) leads to severe leukocytosis in the adult murine epididymis, as detected by histopathological analyses. **(A)** Leukocytes (contained in yellow circles or ovals) infiltrated into the luminal compartment of the *Tgfbr2*^ΔDC^ cauda epididymis. **(B)** Control (*Cre*^−^) cauda epididymis exhibits few somatic cells (yellow circles), as expected. **(C)** A typical case of severe *Tgfbr2*^ΔDC^ epididymal leukocytosis in which leukocytes are abundant both in the periphery of duct profiles and in the luminal space. **(D)** Large portions of epididymis luminal space were occupied by leukocytes and debris (dashed line indicates duct epithelial lining and dotted line indicates area still occupied mostly by sperm). **(E)** Epididymal granulomas (g) were detected in 4 of 12 *Tgfbr2*^ΔDC^ epididymides analyzed. In addition to granulomas, this particular sample exhibited an apparent duct obstruction as sperm accumulated in initial segment (*). **(F)** Large sperm granuloma (g) in *Tgfbr2*^ΔDC^ corpus epididymis. **(G)** Control testis. **(H)** *Tgfbr2*^ΔDC^ testes, including in 3 of 4 *Tgfbr2*^ΔDC^ males with epididymal granulomas, exhibited no signs of histopathology. **(I)** Testicular pathology (arrows and rectangular boxes) localized in seminiferous tubules of 1 of 12 *Tgfbr2*^ΔDC^ males analyzed. The rete testis (†) appears expanded and contains sperm, isolated non-sperm cells and cell clumps. **(J,K)**. Boxed areas from panel **(I)** shown magnified. Tubular histopathology in seminiferous tubules is not concurrent with increased number of interstitial cells (leukocytosis) and seminiferous epithelia detachment appears present (#). *N* = 12 *Tgfbr2*^ΔDC^ males and five control (*Cre*^−^) littermates at age 8–10 weeks old. Scale bars = 50 µm.

Dendritic cells populate the epididymal peritubular and interstitial spaces and, in the initial segment of the epididymis, DCs are also located within the epithelial layer (8, 33). The overall goal of this study was to test whether DCs have a role in maintaining immunotolerance to sperm and, specifically, whether TGFβ signaling in DCs is a factor required for sperm immunotolerance. To begin assessing this, we hypothesized that adult male mice carrying significant TGFβ signaling reduction in DCs specifically (*Tgfbr2*^ΔDC^ males) would exhibit epididymal leukocytosis. Histopathological analyses were derived from *Tgfbr2*^ΔDC^ epididymis and testis, along with the respective littermate control (*Cre*^−^) tissues. Epididymal leukocytosis was detected in all *Tgfbr2*^ΔDC^ samples analyzed (Figure 1). Importantly, leukocyte infiltrations were present also in the luminal spaces, where leukocytes were in direct contact with sperm (Figures 1A,C,D). Frequently affected epididymal portions were the corpus and cauda epididymis (Figures 1C,D,F); however, the initial segment and caput of the *Tgfbr2*^ΔDC^ epididymis also exhibited luminal leukocytosis and granuloma with duct obstruction (Figure 1E). Epididymal granulomas were detected in 4 of 12 *Tgfbr2*^ΔDC^ males analyzed. The *Tgfbr2*^ΔDC^ testes did not present signs of leukocytosis, even in those males with epididymal granulomas (Figures 1G,H). However, in one of these males, the testis exhibited histopathology that appeared to derive from excurrent duct obstruction (Figures 1I–K). These histopathological outcomes suggest that DCs support immunotolerance in the epididymis and that TGFβ signaling in these cells is required for the maintenance of epididymal immunotolerance. It remains unknown whether DCs regulate tolerance in an active tolerogenic manner that is TGFβ signaling dependent. Alternatively, TGFβ signaling in DCs may maintain epididymal immunotolerance by keeping DCs in a passive state, possibly in an immature or non-functional (no antigen presentation) state.

### Adult Males Lacking TGFβ Signaling in DCs and Mature Lymphocytes Do Not Develop Epididymal Leukocytosis

**Figure 2:**
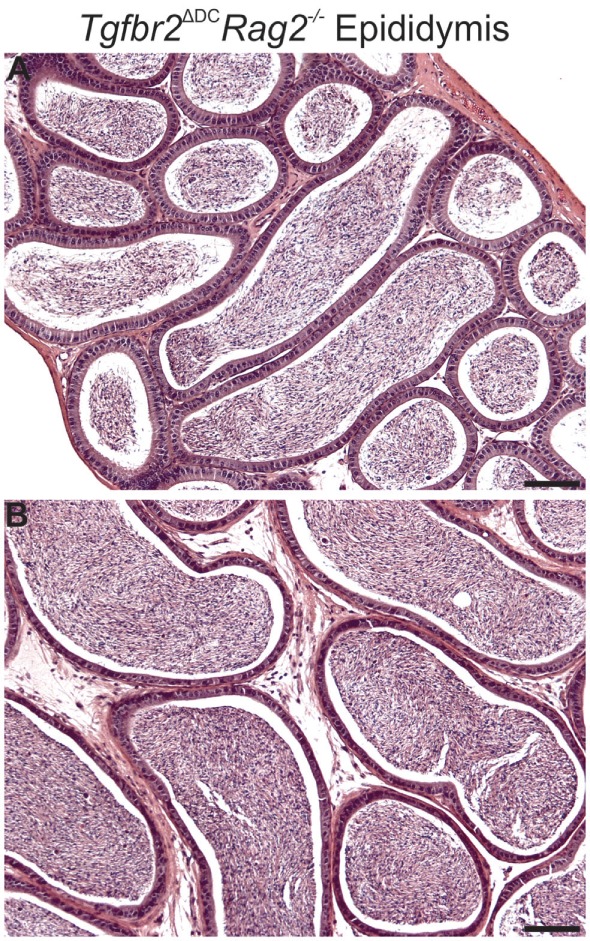
Males lacking both mature lymphocytes and transforming growth factor beta (TGFβ)-signaling in dendritic cells (DCs) do not develop epididymal leukocytosis. Typical images from corpus **(A)** and cauda **(B)** epididymis from *Tgfbr2*^ΔDC^*Rag2^−/−^* males. Abundant sperm are in luminal spaces; however, no inflammatory cell infiltrations are present. *N* = 6 *Tgfbr2*^ΔDC^*Rag2^−/−^* males and two control (*Cre*^−^) littermates at age 8–10 weeks. Scale bars = 50 µm.

Mice carrying germline deletion of recombination activating gene 2 (*Rag2^−/−^*) do not acquire mature T and B cells (34), and this murine model is extensively employed in testing the participation of lymphocytes in various inflammatory contexts. We generated *Tgfbr2*^ΔDC^ males in the *Rag2^−/−^* background (*Tgfbr2*^ΔDC^*Rag2^−/−^*) and examined their epididymis and testis by histology. Leukocytosis was not detected in any of the adult (8–10 weeks old) *Tgfbr2*^ΔDC^*Rag2^−/−^* epididymides (Figure 2) or testes (data not shown). These outcomes suggest that the immune response that impacts the epididymis with severe leukocytosis (when males carry disruption of TGFβ signaling in DCs) requires the participation and chemotaxis of T and/or B cells to this tissue.

### Flow Cytometry Detects Leukocytosis in *Tgfbr2*^ΔDC^ Epididymis but Not Testis; T_regs_ Are Increased in Both

**Figure 3:**
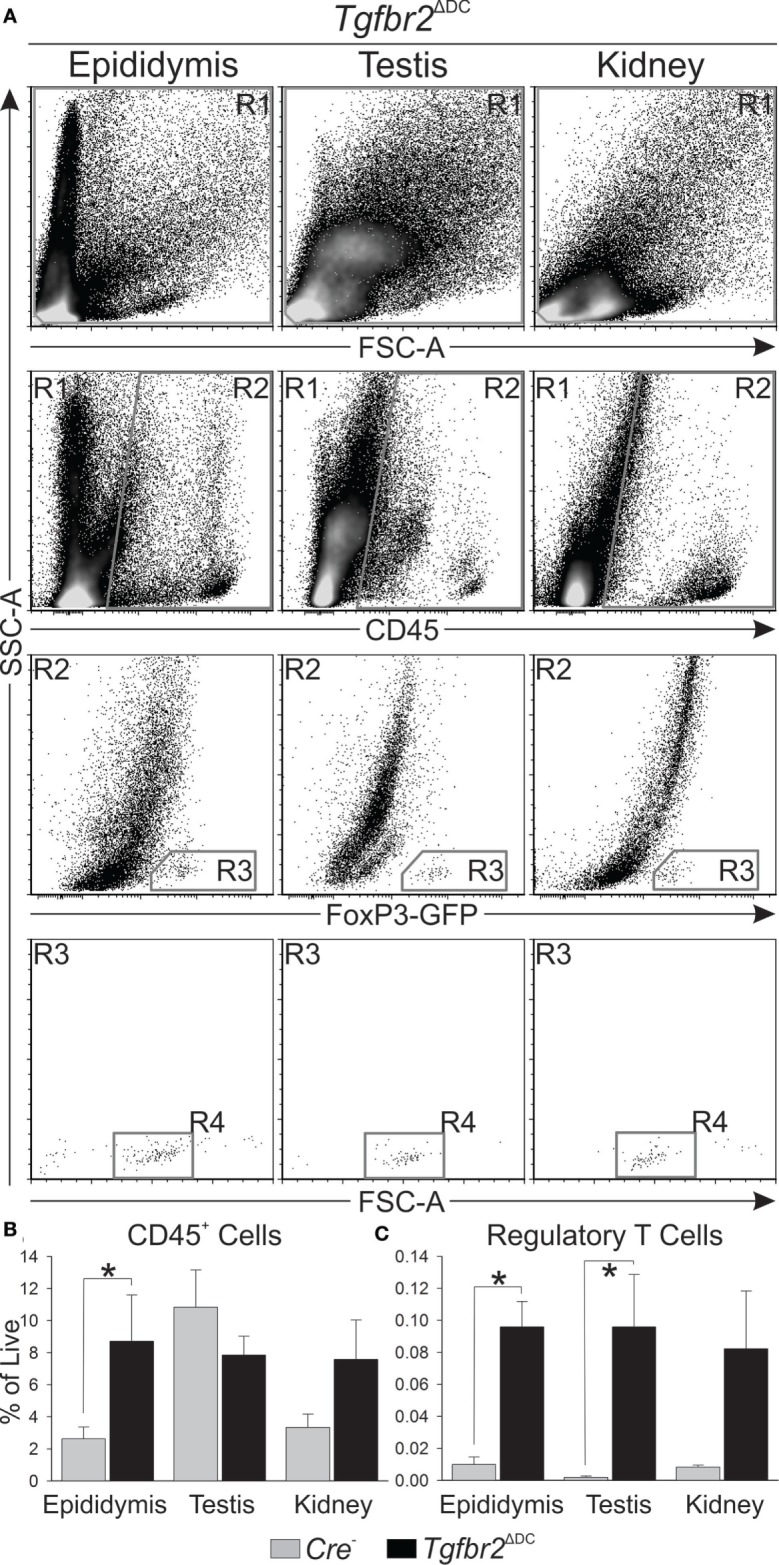
Flow cytometric analyses show leukocytosis is greatest in the epididymis when dendritic cells (DCs) lack transforming growth factor beta (TGFβ) signaling. **(A)** Typical flow cytometry results derived from epididymis, testis, and kidney, and gating strategy employed are shown. All cell suspensions were exposed to a cell viability dye and cells in R1 were separated by a “live gate” (data not shown) prior to definition of CD45^+^ cell numbers by R2. Regulatory T cells (T_regs_) were first gated by R3 and T_reg_ numbers were ultimately defined by R4. **(B)** Summary of all CD45^+^ cell quantitation shows the *Tgfbr2*^ΔDC^ epididymis exhibits the greatest leukocytosis, while the *Tgfbr2*^ΔDC^ testis does not seem affected. Observations included isotype control antibody stains (data not shown), which were employed in data analyses as described in Section “Materials and Methods.” **(C)** T_regs_, detected at low numbers in control tissues, were significantly increased in *Tgfbr2*^ΔDC^ epididymis and testis. Data are expressed as “% of Live,” as in panel **(B)**. *N* = 5 (pairs of *Cre*^−^ and *Tgfbr2*^ΔDC^ littermate males, age 9–10 weeks); *p* ≤ 0.05. A total of 100,000 events per sample were recorded.

To test for leukocytosis in *Tgfbr2*^ΔDC^ male tissues at the molecular level, flow cytometric analyses were conducted to quantify CD45^+^ cells (leukocytes) in control (*Cre*^−^) and *Tgfbr2*^ΔDC^ epididymis and testis. Kidneys from the same mice, serving as a reference tissue of similar embryological origin that is free of sperm, were also analyzed. In addition, DCs are known to regulate peripheral tolerance by inducing differentiation of T_regs_ (35) and *Tgfbr2*^ΔDC^ mice are known to exhibit altered T_reg_ phenotype (25). Expression of forkhead box P3 (FoxP3) is essential for T_reg_ development and function (36, 37) and is recognized as an unambiguous T_reg_ marker (38). To facilitate T_reg_ analyses in *Tgfbr2*^ΔDC^ mice, a GFP knock-in allele (FoxP3-GFP) was bred in, and both the controls (*Cre*^−^) and *Tgfbr2*^ΔDC^ mice tested expressed GFP downstream from T_reg_ specific FoxP3 expression. Thus, in these experiments, leukocytes were identified and quantified by immunolabeling with monoclonal anti-mouse CD45 directly conjugated to PE-Cy5, while T_regs_ were identified and quantified *via* detection of FoxP3-induced GFP. Spleen cell suspensions were also tested to unequivocally identify GFP^+^ cells (T_regs_) exhibiting morphology (forward and side scatters) consistent with those of T cells and for use in the generation of flow cytometry compensation matrices (GFP and live/dead signals, data not shown). Outcomes indicated that the *Tgfbr2*^ΔDC^ epididymis contains a significantly more abundant CD45^+^ population, whereas the *Tgfbr2*^ΔDC^ kidney exhibits a suggestive increase in CD45^+^ cells that did not reach statistical significance (Figures 3A,B). In the *Tgfbr2*^ΔDC^ testis, the quantity of CD45^+^ cells was unchanged or only slightly decreased (Figure 3B). T_regs_ were significantly increased in both the *Tgfbr2*^ΔDC^ epididymis and testis (Figure 3C). Interestingly, despite harboring the largest CD45^+^ population on average, the testis presented the smallest T_reg_ population among the *Cre*^−^ tissues tested. These flow cytometry outcomes quantitatively frame what the histopathology outcomes had suggested. The adult *Tgfbr2*^ΔDC^ epididymis contains the most leukocytes, whereas the *Tgfbr2*^ΔDC^ testis occupies the opposite end of the spectrum with no changes (or a non-significant decrease) in the CD45^+^ population. Importantly, these results suggest that the *Tgfbr2*^ΔDC^ epididymis and testis contain significant expansions in their T_reg_ compartments. In these initial experiments, the numbers of T_regs_ detected in control testis samples were very low. This was initially assumed to be due, at least in part, to the fact that the total number of events recorded per sample was 10^5^. This and the known role of T_regs_ in immunotolerance prompted a dedicated set of experiments to quantify T_regs_ in control and *Tgfbr2*^ΔDC^ male tissues.

### The *Tgfbr2*^ΔDC^ Epididymis and Testis Exhibit T_reg_ Increases

**Figure 4:**
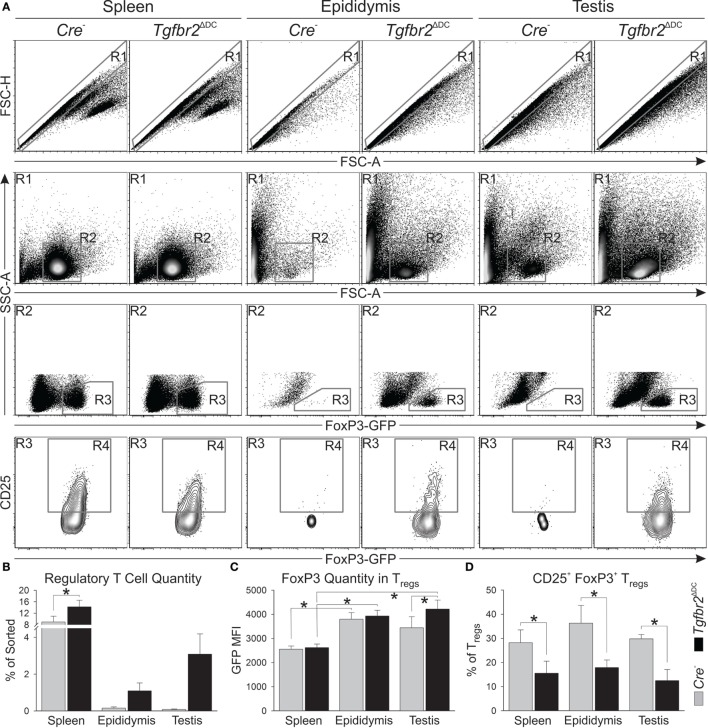
Loss of transforming growth factor beta (TGFβ) signaling in dendritic cells (DCs) leads the epididymis and testis to contain regulatory T cells (T_regs_) at numbers much greater than at steady state. **(A)** Typical flow cytometry outcomes from CD4^+^ sorted splenic, epididymal, and testicular cell suspensions, and the gating strategy used are shown. T_reg_ numbers and the estimates of *FoxP3* expression were defined with R3. CD25^+^FoxP3^+^ T_regs_ were quantified with R4. **(B)** T_reg_ increases were detected in all the *Tgfbr2*^ΔDC^ tissues tested. **(C)** Estimates of *FoxP3* expression in T_regs_ from the tissues tested. **(D)** CD25^+^FoxP3^+^ T_regs_ were significantly reduced in all *Tgfbr2*^ΔDC^ tissues tested. *N* = 3 (pairs of *Cre*^−^ and *Tgfbr2*^ΔDC^ littermate males, age 9–10 weeks); *p* ≤ 0.05. All samples were recorded to exhaustion and the average number of events in R1 across all samples was 114,000.

Regulatory T cells were shown to be required for suppression of orchitis in a murine model of vasectomy pathology (39) and our initial flow cytometry results (Figure 3) showed the *Tgfbr2*^ΔDC^ testis does not exhibit leukocytosis, but a significant increase in T_regs_. Furthermore, the phenotype of *Tgfbr2*^ΔDC^ T_regs_ from spleen and mesenteric lymph nodes is altered in regard to expression of interleukin-2 (IL-2) receptor α-chain (CD25) and other parameters (25). Therefore, we conducted an additional set of experiments to identify T_reg_ phenotypic differences. Cell suspensions derived from all epididymal and testicular tissues, along with 50 million splenocytes from each of 3 control and 3 *Tgfbr2*^ΔDC^ males were sorted magnetically based on CD4 expression. CD4^+^ cell isolates were then subjected to flow cytometry detecting *FoxP3*-induced GFP fluorescence and CD25. In flow cytometry, forward scatter-height data were also acquired, thus allowing for gating and analyses of singlet cells (Figure 4A, gate R1). The control (*Cre*^−^) epididymis and testis contained low T_reg_ numbers (Figure 4A, gate R3 and Figure 4B). However, T_regs_ were markedly increased in the *Tgfbr2*^ΔDC^ epididymis and testis (Figures 4A,B). *FoxP3* expression in male tissue T_regs_ was significantly greater than in splenic T_regs_, and significantly greater in *Tgfbr2*^ΔDC^ testicular T_regs_ than in their *Cre*^−^ counterparts (Figure 4C). The proportion of CD25^+^ T_regs_ was significantly reduced in all *Tgfbr2*^ΔDC^ tissues tested, compared with *Cre*^−^ controls (Figure 4D). Together, these results suggest that the epididymis and testis contain, at steady state, relatively few T_regs_ but that these T_reg_ compartments expand by several folds in a pro-inflammatory scenario such as that of the *Tgfbr2*^ΔDC^ male tract. It remains to be tested whether these T_reg_ populations exhibit suppressor function and whether T_reg_ expansions regulate (suppress) inflammatory leukocytosis in the *Tgfbr2*^ΔDC^ testis and epididymis.

### Adult *Tgfbr2*^ΔDC^ Males Exhibit an Immune Response That Targets Sperm Specifically

**Figure 5:**
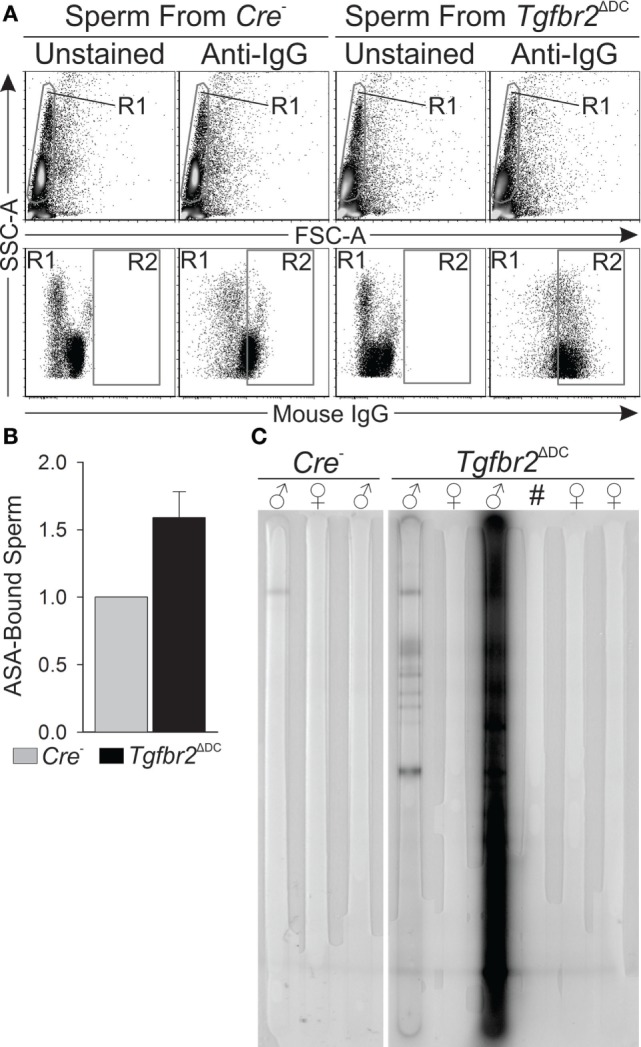
Adult *Tgfbr2*^ΔDC^ males exhibit sperm-specific immune response. **(A)** Typical flow cytometry outcomes that detected immunoglobulins (IgGs) bound to sperm [antisperm antibodies (ASAs)]. Sperm (gated in R1) were determined to be “positive” or “ASA-bound” if their fluorescence signal was intense enough to place a particular sperm cell within R2. **(B)** Summary of IgG-bound sperm quantifications. In each paired observation, the quantity (% of all sperm) of IgG-bound sperm from the *Cre*^−^ male was taken as baseline (value = 1) and the mean fold-change of IgG-bound sperm from *Tgfbr2*^ΔDC^ littermates is shown. *N* = 4, and 100,000 events recorded per sample. **(C)** Immunoblot-based assay that detected free ASAs (IgGs) in sera. Signals were generated where ASAs in sera bound to sperm antigens immobilized on the membrane. Signals are multiple and intense for the second *Tgfbr2*^ΔDC^ male shown. All samples tested by this method are shown. All mice tested were ages 8–10 weeks.

Figure 1 shows that leukocytes infiltrate into the *Tgfbr2*^ΔDC^ epididymal lumen and, although this suggests that the *Tgfbr2*^ΔDC^ epididymal immune response is sperm targeted, more definitive evidence of a sperm-specific immune response was not available. To address this, we tested for ASAs both bound to sperm and free in sera. ASAs bound to sperm were quantified by incubating sperm-rich cells suspensions in the presence or absence of a fluorophore-conjugated anti-mouse immunoglobulin G (IgG) antibody and subsequent flow cytometry. Results from this approach reveal that sperm from *Tgfbr2*^ΔDC^ mice exhibit a bound ASA signal that is, on average, 60% greater than that presented by sperm from *Cre*^−^ littermates (Figures 5A,B). In an immunoblot-based assay, we tested for presence or absence of free ASAs in sera from adults and assessed the specificity of the signals by also testing sera from virgin females. Sera from *Tgfbr2*^ΔDC^ males generated abundant ASA signals (Figure 5C). These results demonstrate unequivocally that *Tgfbr2*^ΔDC^ males generate an immune response against sperm, supporting the overarching hypothesis of this study, i.e., that TGFβ signaling in DCs is required for immunotolerance to sperm. Outcomes presented in Figures 1–5 suggest that epididymal DCs, once deprived of TGFβ signaling, will initiate a sperm-specific immune response that translates onto epididymal inflammatory leukocytosis and ASAs. Results in Figure 4 suggest the epididymal and testicular T_reg_ expansions occur as an attempt to maintain immunotolerance to sperm.

### Distinct Epididymal and Testicular Transcriptomes in *Tgfbr2*^ΔDC^ Males

**Figure 6:**
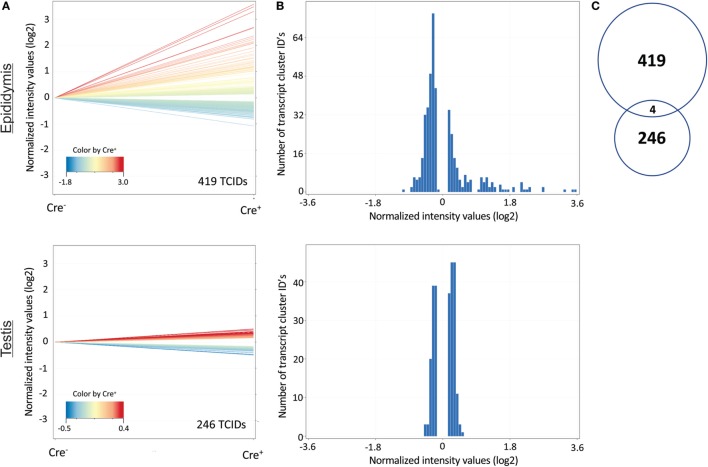
Distinct changes in the transcriptional profiles of the epididymis and testis of *Tgfbr2*^ΔDC^ mice. **(A)** Genes (denoted as transcript cluster IDs or TCIDs) were identified as differentially expressed in *Cre*^+^ mice by moderated *t*-test with Westfall–Young multiple testing correction (*p* < 0.05; *N* = 3). **(B)** Histograms depicting the patterns of altered gene expression in the two tissues, combining the magnitude of change (binned into 50 fold-change categories, *X*-axis) and the number of TCIDs (*Y*-axis). **(C)** Venn diagram demonstrating negligible overlap (4 TCIDs) between the sets of differentially regulated genes in the epididymis (419 TCIDs) and testis (246 TCIDs).

**Figure 7:**
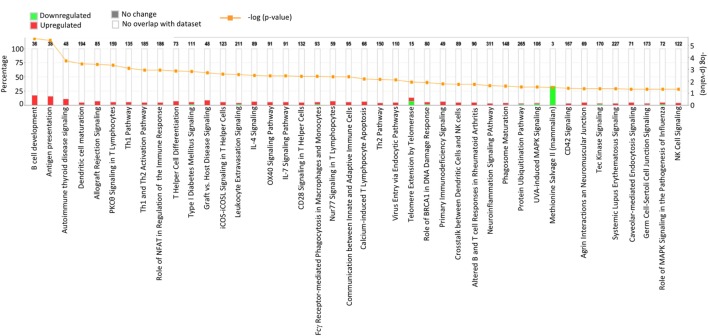
Ingenuity Pathway Analysis (IPA) of the 419 transcript cluster IDs (TCIDs) significantly altered in *Tgfbr2*^ΔDC^ epididymis. The identified pathways with overrepresented TCIDs are depicted on the *X*-axis. Left *Y*-axis denotes the percentage of genes among the 419 TCIDs in the total number of genes in any given category (total number of genes in each pathway is directly above each stacked bar). Right *Y*-axis (yellow line) depicts negative logarithm of the *p*-value for each pathway. The number of upregulated genes in *Cre*^+^ mice is depicted in red, while downregulated genes are shown in green.

**Figure 8:**
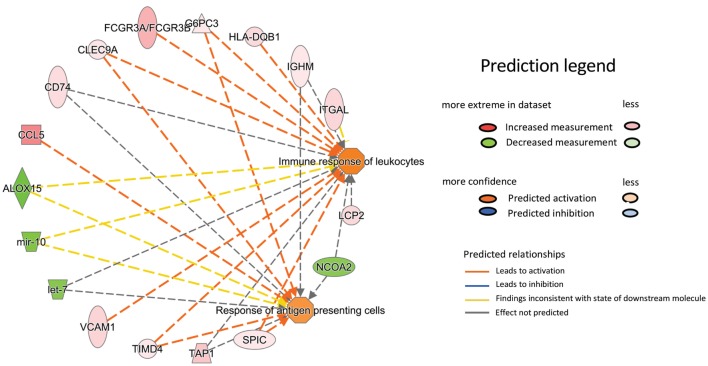
Ingenuity Pathway Analysis network representation of two prominently overrepresented pathways in the epididymis of the *Tgfbr2*^ΔDC^ mice with high relevance to the model of autoimmunity: response of antigen-presenting cells (*p* = 4.17E−005) and immune response of leukocytes (*p* = 2.5E−005). The relationship and direction of change are indicated as upregulated in *Cre*^+^ (shades of red) or downregulated in *Cre*^+^ (shades of green).

**Figure 9:**
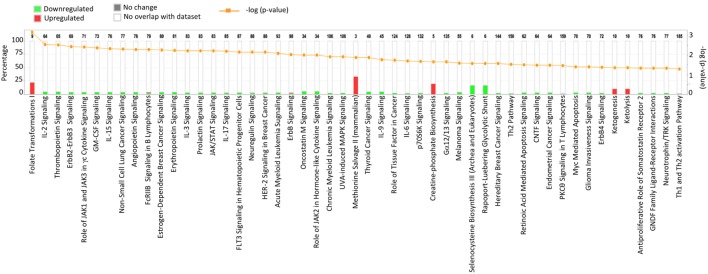
Ingenuity Pathway Analysis of the 246 transcript cluster IDs (TCIDs) significantly altered in *Tgfbr2*^ΔDC^ testis. Identified pathways with overrepresented TCIDs are depicted on the *X*-axis. Left *Y*-axis denotes the percentage of genes among the 246 TCIDs in the total number of genes in any given category (total number of genes in each pathway is directly above each stacked bar). Right *Y*-axis (yellow line) depicts negative logarithm of the *p*-value for each pathway. The number of upregulated genes in *Cre*^+^ mice is depicted in red, while downregulated genes are shown in green.

Results acquired to this point indicated sharp differences between the *Tgfbr2*^ΔDC^ epididymis and testis at the histopathological and immunological levels. To test for whole-organ transcriptional differences, we derived microarray-based gene expression analysis from *Cre*^−^ and *Cre*^+^ tissues (littermates). As depicted in Figure 6, consistent with the inflammatory response detected in the epididymis, significant changes in gene expression were observed in this tissue: 419 transcript cluster IDs (TCIDs) were differentially regulated under stringent statistical parameters (Figure 6A). In the testis, 246 TCIDs were differentially expressed, and this expression was within a narrower range than that of the epididymis, as can be noted also in the scales for the fold-change color diagrams (Figure 6A). Figure 6B represents these transcriptional differences further by indicating the number and distribution of over- and under-expressed genes along the fold-change ranges of each tissue type. Importantly, there was a negligible overlap between the differentially regulated epididymal and testicular genes (only four TCIDs in common, Figure 6C), providing evidence of very different transcriptional responses to the loss of DC-mediated immunotolerance in those two tissues. IPAs were conducted using the epididymal (419 TCIDs) and testicular (246 TCIDs) sets of regulated genes. Two conclusions are substantiated by the outcomes of IPA analyses. In the epididymis, consistent with the observed leukocytosis, the genes upregulated were associated with inflammatory response pathways such as B cell development, antigen processing and presentation, DC maturation, both T_h_1 and T_h_2 cell activation, and others (Figure 7). Two of the most statistically significant biofunction categories identified by IPA were response of antigen-presenting cells and immune response of leukocytes, which were assessed further by network analysis (Figure 8). In the testis, intriguingly, genes and pathways potentially involved with inflammatory response (IL-2, GM-CSF, IL-15, IL-3, JAK/STAT, IL-17, IL-9, and IL-6) were predominantly downregulated (Figure 9). Furthermore, the testis displayed upregulation of genes and pathways involved in metabolism, such as folate transformation, methionine salvage, ketogenesis, and ketolysis (Figure 9). As discussed subsequently, this outcome may constitute the first evidence for a testicular metabolic-based mechanism for maintenance of immunotolerance.

## Discussion

Results reported in this study reveal that loss of TGFβ signaling in DCs adversely impacts male reproductive tract health by inducing severe epididymal pathology that is characterized by leukocytosis with granulomas, generation of ASAs and upregulation of pro-inflammatory pathways. Importantly, these data suggest that TGFβ signaling in DCs is a factor required for immunotolerance to sperm in the epididymis and that testicular immunosuppressive factors cannot maintain tolerance in the epididymis when TGFβ signaling in DCs is disrupted.

Although it is possible that TGFβ signaling may simply keep epididymal DCs at an immature and non-functional state, data now available suggest otherwise. Greater than 88% of all CD11c^+^ cells in C57B6 wild-type epididymis express MHCII, and ~37% of these cells express the activation markers CD80 or CD86 (8), suggesting that nearly all epididymal DCs are enabled with antigen presentation capacity and that one-third of this population is activated. As we showed here, abrogation of TGFβ signaling led to epididymal leukocytosis that requires T and/or B cells (Figures 1 and 2). Therefore, the available data suggest that TGFβ signaling induces tolerogenic activity in DCs of the epididymis, an organ that contains, as known to date, the largest DC population of the male tract.

Our results showed that control mice exhibit low T_reg_ numbers in epididymis and testis while the respective *Tgfbr2*^ΔDC^ organs presented increased T_reg_ numbers and significantly greater *FoxP3* expression in T_regs_. Moreover, the *Tgfbr2*^ΔDC^ testes did not exhibit detectable leukocytosis, despite severe leukocytosis and/or granulomas in epididymides. Both the origin and the functional status of T_regs_ in *Tgfbr2*^ΔDC^ epididymis and testis remain unknown. Testicular M2 macrophages of the rat are able to induce T_reg_ differentiation *in vitro* (40). Thus, it is possible that M2 macrophages account for at least a portion of the detected T_reg_ increases in the *Tgfbr2*^ΔDC^ testis. Testicular DCs can actively promote antisperm immunity as shown with the rat experimental orchitis model (41), and DCs in the *Tgfbr2*^ΔDC^ testis are expected to be pro-inflammatory. Despite this, the *Tgfbr2*^ΔDC^ testis exhibited no leukocytosis while showing downregulation of genes and pathways associated with inflammation, which strongly suggests that there are robust immunosuppressive mechanisms actively functioning in this organ to maintain immunotolerance. As shown with the mouse vasectomy model, T_regs_ can maintain the testis free of inflammation while vasectomy-induced epididymitis is present (39). Therefore, T_regs_ can contribute to immunotolerance in the *Tgfbr2*^ΔDC^ testis. Additional testicular immunosuppressive factors may be contributing to the *Tgfbr2*^ΔDC^ testicular phenotype as well. M2 macrophages themselves are immunosuppressive and abundant in the testis (40, 42). Sertoli cells can secrete factors that have direct immunosuppressive activity (43–45). Our transcriptional analyses suggest an additional and novel mechanistic element in that the testis exhibited altered metabolism when antisperm immunity is present. Specifically, IPA analysis showed that the only upregulated pathways were metabolic. Upregulation of ketogenesis and ketolysis suggests that the *Tgfbr2*^ΔDC^ testis may have switched to using fat as fuel instead of carbohydrates. This switch, as proposed by others, is exactly what allows “a cell” to downregulate MHC expression and co-stimulatory molecules and therefore become less visible to the immune system (46). Ultimately, additional investigation is needed to address this possibility and to define the origin and function of *Tgfbr2*^ΔDC^ testicular and epididymal T_regs_ and other immunosuppressive factors potentially functioning in the *Tgfbr2*^ΔDC^ testis.

The results reported in this study enhance our understanding of male tract immunotolerance and are potentially significant for human clinical research. ASAs and leukocytospermia are prevalent clinical signs detected in infertile men, and ASAs are demonstrated causal factors of human male infertility (47, 48). Results reported here suggest that the primary etiology of both these pathologies can be totally independent of infection, prior male duct obstruction, vasectomy, or trauma; and can originate at—and be restricted to—the epididymis. Finally, the unique immunosuppressive capacity of the male tract is been recognized as a strong candidate justification to how Zika, HIV, and Ebola viruses escape complete clearance and remain transmissible in the epididymis (Ebola) or testis (49, 50).

The outcomes reported here appear to also highlight the importance of peripheral—and in the case of the epididymis and sperm antigens, DC-mediated—mechanisms of tolerance for immunotolerance to sperm, male reproductive tract integrity, and function. Disruption of central tolerance by global ablation of the autoimmune regulator in mice does not induce testicular lymphocytic infiltrations, and ASAs or epididymal pathology have not been demonstrated (51–53).

Ultimately, data reported here suggest that TGFβ signaling in DCs is required for immunotolerance to sperm in the epididymis, as mice lacking TGFβ signaling in DCs are shown to develop severe epididymal inflammatory pathology and ASAs.

## Ethics Statement

All mice used in the reported study were generated and utilized under the guidelines of an animal protocol approved by the Kansas State University Institutional Animal Care and Use Committee (IACUC; to Dr. Pierucci-Alves), and by the University of Arizona IACUC (to Dr. Kiela).

## Author Contributions

FP-A conceived study. FP-A and PK planned and executed experimental work, data analyses, and wrote the manuscript. MM-K, SF, and BS contributed to experimental work and/or resources needed and/or data analyses and/or manuscript writing.

## Conflict of Interest Statement

The authors declare that this research was conducted in the absence of any commercial or financial relationships that could be construed as a potential conflict of interest.
